# Trends in Atrial Fibrillation and Ablation Therapy During the Coronavirus Disease 2019 Pandemic

**DOI:** 10.19102/icrm.2024.15074

**Published:** 2024-07-15

**Authors:** Anmol Johal, Joseph Heaton, Abbas Alshami, Ndausung Udongwo, Steven Imburgio, Anton Mararenko, Brett Sealove, Jesus Almendral, Jeffrey Selan, Riple Hansalia

**Affiliations:** 1 Department of Medicine, Jersey Shore University Medical Center, Neptune City, NJ, USA; 2 Department of Medicine, Division of Cardiology, Jersey Shore University Medical Center, Neptune City, NJ, USA

**Keywords:** Ablation, arrhythmia, atrial fibrillation, COVID-19

## Abstract

The coronavirus disease 2019 (COVID-19) pandemic affected many aspects of health care and continues to have an impact as waves of COVID-19 cases re-emerge. Many procedures were negatively impacted by the pandemic, and management was primarily focused on limiting exposure to the virus. We present an analysis of the National Inpatient Sample (NIS) to delineate how COVID-19 affected atrial fibrillation (AF) ablation. The NIS was analyzed from 2017–2020 in order to determine the pre- and intra-pandemic impacts on AF ablation procedures. Admissions were identified using the International Classification of Diseases, 10th Revision, Clinical Modification codes with a primary diagnosis of AF (ICD-10 CM code I48.0, I48.1, I48.2, or I48.91). Admissions were also assessed for the use of cardiac ablation therapy. Comorbidity diagnoses were identified using the Elixhauser comorbidity software (Agency for Healthcare Research and Quality, Healthcare Cost and Utilization Project, Rockville, MD, USA); additional ICD-10 codes for diagnoses and procedures used are also provided. The primary outcome of our study was the trend in ablation therapy during AF admissions. Secondary outcomes included health care disparities, inpatient mortality, and length of stay. Ablation therapy was used in 18,885 admissions in 2020, compared to the preceding 3-year average of 20,103 (adjusted Wald test, *P* = .002). Multivariate logistic regression revealed a greater likelihood of undergoing ablation therapy (odds ratio, 1.24; 95% confidence interval, 1.10–1.40; *P* < .001) among 2020 admissions compared to 2017 admissions. Inpatient mortality increased in 2020 compared to the preceding average; however, the difference was not significant. The procedural volume of ablation for AF saw a decrease in 2020; however, surprisingly, more patients were likely to undergo ablation during 2020.

## Background

The coronavirus disease 2019 (COVID-19) pandemic significantly affected health care usage, including emergency cardiovascular procedures.^1^ Though COVID-19 is primarily a respiratory illness, it also affects the cardiovascular system directly.^2^ Current evidence is still limited; however, suggested pathophysiologic processes have described the mechanism of severe acute respiratory syndrome coronavirus 2 (SARS-CoV-2) causing inflammation and damaging cardiac cells in humans.^3^ Animal and in vitro studies have suggested that electrical disturbances may be related to a direct interaction with angiotensin-converting enzyme-2 receptors and alteration of action potentials related to inflammatory states.^4^

Management of atrial fibrillation (AF) during the COVID-19 pandemic was augmented by contemporary practice guidelines, which focused on medical management techniques.^5^ Anecdotal evidence was available to support the feasibility of ablation therapy during the pandemic, although the evidence in question was limited.^6^ Consequently, the recommendations for using ablation therapy in the background of COVID-19 were unclear. Previous studies have also revealed health care disparities in managing AF,^7^ and the confounding effect of COVID-19 is not well characterized.

### Objective

We endeavored to describe national trends relating to the effect of COVID-19 on AF admissions and the use of ablation therapy.

## Methods

The Healthcare Cost and Utilization Project’s (HCUP) National Inpatient Sample (NIS) is a nationally representative all-payer, claims-based inpatient discharge sampling database representing a stratified 20% sample of all non-federal US hospitals, describing >35 million annual hospitalizations in the United States.^8^ Admissions were identified between 2017–2020 using International Classification of Diseases, 10^th^ Revision codes. Admissions were included if the patient was aged ≥18 years and admitted non-electively with a primary diagnosis of AF (ICD-10-CM code I48.0, I48.1, I48.2, or I48.91). Admissions were also assessed for the use of cardiac ablation therapy. Comorbidity diagnoses were identified using the Elixhauser comorbidity software^9^; additional ICD-10 codes for diagnoses and procedures used are provided in **Supplementary Table 1**. The primary outcome of our study was the trend in ablation therapy during AF admissions. Secondary outcomes included health care disparities, inpatient mortality, and length of stay.

Statistical analysis methods followed the HCUP’s best practices, including national estimates using discharge weights, observations identified as hospitalization events, and inferential statistical reporting due to the complex survey methodology. Categorical values are represented as percentages and compared using the chi-squared test. Continuous variables are represented as means and compared using the adjusted Wald test. Multivariable regression analyses analyzed the odds of undergoing ablation therapy, inpatient mortality, and length of stay. All statistical analyses were performed using Stata 17 (StataCorp LLC, College Station, TX, USA), using the “svy” function to account for complex survey methodology with weighting. Statistical significance was determined by an α (*P*) value of .05. Institutional review board approval is not required for studies using the NIS, as it is a “limited dataset” exempted from Health Insurance Portability and Accountability Act privacy regulations. The present study was conducted in alignment with the principles of the Declaration of Helsinki and the EQUATOR Network’s Strengthening the Reporting of Observational Studies in Epidemiology guidelines.^10^

## Results

During the period of 2017–2020, 1,510,465 admissions met the inclusion criteria. The average age at admission was 70.75 ± 12.6 years, with 3.92 comorbidities present. Women represented 51.23% of all admissions, and 81.97% were White. Baseline characteristics are provided in **Table 1**.

**Figure 1:**
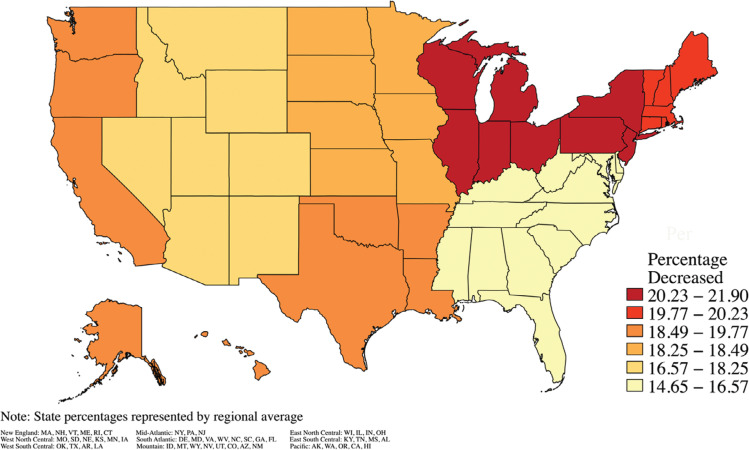
Trends in ablation therapy use. Ablation therapy use in 2020 compared to the preceding 3-year average. States are represented by National Inpatient Sample–designated regional averages.

**Figure 2:**
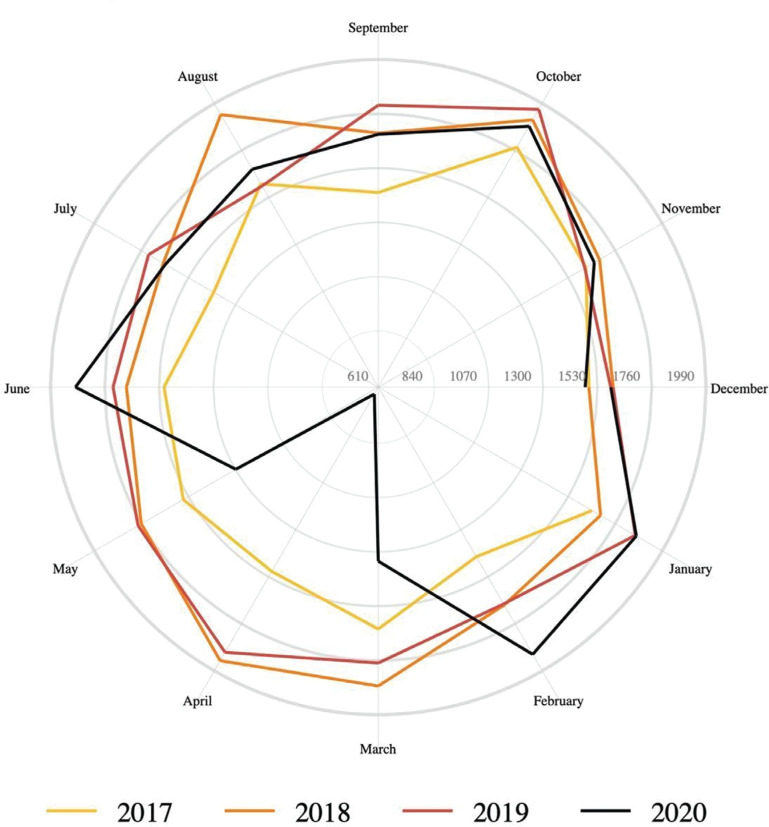
Monthly trends in ablation therapy use. Ablation therapy use during 2017–2020 by month.

In 2020, 321,535 admissions were identified, compared to the preceding 3-year average of 396,310. Ablation therapy was used in 18,885 admissions in 2020, compared to the preceding 3-year average of 20,103 (adjusted Wald test, *P* = .002). Regional changes in the procedural volume during 2020 are presented in **Figure 1**. The monthly procedural volume from 2017–2020 is presented in **Figure 2**. Multivariate logistic regression **(Supplementary Table 2)** revealed a greater likelihood of undergoing ablation therapy (odds ratio [OR], 1.24; 95% confidence interval [CI], 1.10–1.40; *P* < .001) among 2020 admissions compared to 2017 admissions.

During admissions for AF, ablation was performed less frequently in Black, Hispanic, and Asian or Pacific Islander patients compared to White patients (*P* < .05, all; **Supplementary Table 2**). However, there were no changes in trends for any race during the observed period (all *P* > .05). Women were also less likely to undergo ablation (OR, 0.73; 95% CI, 0.70–0.76; *P* < .001) compared to men. Admitted patients were more likely to undergo ablation therapy if their household income was between the 51^st^ and 75^th^ percentiles (OR, 1.15; 95% CI, 1.08–1.23; *P* < .001) or above the 75^th^ percentile (OR, 1.35; 95% CI, 1.25–1.45; *P* < .001).

The inpatient mortality for patients admitted for AF increased in 2020 compared to the preceding average (1.07% vs. 0.87%, respectively), albeit in a manner not found to be significantly associated when using logistic regression modeling (*P* = .087; **Supplementary Table 3**). The use of ablation therapy was associated with a lower risk of inpatient mortality (OR, 0.64; 95% CI, 0.52–0.80; *P* < .001). The length of stay showed a 0.08-day (95% CI, −0.12 to −0.03; *P* = .001) reduction in 2020 compared to 2017.

## Discussion

This nationally representative study revealed that the COVID-19 pandemic was associated with an overall decrease in AF admissions and ablation use. This finding aligns with those of other studies that have detailed the decreases in health care use during this time, including with life-saving procedures.^1^ The decrease in admissions identified may have been influenced by hesitancy among patients to seek medical care, early mortality due to COVID-19, and patients being admitted due to any other primary cause.

The likelihood of undergoing ablation was significantly higher during 2020 than in preceding years. The exact cause of this trend is difficult to determine and is likely multifactorial in nature. One potential cause may have been related to AF episodes refractory to medical management due to persistent provocation from SARS-CoV-2. Additionally, due to limited outpatient availability during the pandemic, patients requiring ablation therapy may have required an inpatient admission to undergo the procedure. There may have also been an inherent hesitancy to avoid performing AF ablation during the COVID-19 pandemic in 2020 due to risk and provider preference.

Several disparities in the use of cardiac ablation therapy were found during this study. Minority race, female sex, and earning less than the median national income were all independently associated with a lower likelihood of undergoing cardiac ablation therapy. This finding is consistent with results of previous literature,^7^ which described marginalized groups disproportionately affected by cardiac disease.^11^ The exact causes are unclear but may be sequelae of disparities, such as being less likely to undergo diagnostic testing^12^ or be referred for therapy,^13^ or because patients of marginalized groups may be more likely to refuse a medically necessary procedure.^14^

Our study showed that the COVID-19 pandemic affected AF admissions and the use of cardiac ablation therapy. Despite a lower incidence of both, outcomes related to the management of AF were largely unchanged. Specifically, no clinically significant differences in the length of stay or inpatient mortality were found. Nevertheless, health care disparities related to AF management were also unchanged, leaving an opportunity for improvement in the future.

### Limitations

The inherent nature of the database limits this study. Use of the NIS is limited due to the lack of complete medication, imaging, or other patient data. Outpatient and elective procedures are also not accounted for. Diagnoses were identified using ICD-10 codes and may be affected by misidentification or under-identification. Admissions were identified by their primary diagnosis, which may have contributed to fewer patients being identified as undergoing cardiac ablation therapy in the setting of AF if AF was not listed as the first diagnosis. Residual confounders may be missed due to the lack of granular information, including medications and laboratory and electrocardiogram results. Causation cannot be determined due to the retrospective observational nature of this study. The independent effect of COVID-19 on outcomes also could not be accurately determined due to the Centers for Disease Control and Prevention’s restrictive COVID-19 coding policies during the initial pandemic onset.^15^

## Conclusion

The COVID-19 pandemic was associated with fewer admissions for AF and the use of ablation therapy. However, patients were more likely to undergo ablation therapy in 2020 when admitted for AF. Minority race, female sex, and lower economic status were associated with decreased use of ablation, and the pandemic did not influence this trend. More research is needed to understand the impact and management of AF in the presence of COVID-19.

## Figures and Tables

**Table 1:** Baseline Characteristics of Patients Admitted with Atrial Fibrillation

	2017–2019 Mean	2020	Total	Observations	*P* Value
Age (mean), years	70.75 (0.04)	70.77 (0.07)	70.75 (0.03)		<.001
Elixhauser comorbidities (mean)	3.87 (0.01)	4.11 (0.01)	3.92 (0.01)		<.001
Female sex	51.41 (0.12)	50.53 (0.22)	51.23 (0.10)	773,710	<.001
Race					
White	81.92 (0.24)	82.16 (0.41)	81.97 (0.20)	1,211,145	.392
Black	7.97 (0.13)	8.21 (0.24)	8.02 (0.11)	118,525	
Hispanic	6.12 (0.15)	5.64 (0.24)	6.02 (0.12)	88,960	
Asian or Pacific Islander	1.54 (0.05)	1.49 (0.09)	1.53 (0.04)	22,640	
Native American	0.38 (0.03)	0.43 (0.04)	0.39 (0.02)	5770	
Other	2.07 (0.08)	2.07 (0.13)	2.07 (0.07)	30,530	
CHA_2_DS_2_-VASc score ≥ 2 points	86.49 (0.08)	87.09 (0.15)	86.61 (0.07)	1,308,260	.001
Hypertension	79.72 (0.11)	80.86 (0.21)	79.96 (0.10)	1,207,800	<.001
Diabetes	28.25 (0.11)	28.95 (0.21)	28.40 (0.10)	428,900	.003
Hyperlipidemia	51.60 (0.16)	54.55 (0.29)	52.22 (0.14)	788,820	<.001
Obesity	22.11 (0.14)	24.83 (0.27)	22.69 (0.12)	342,735	<.001
Peripheral vascular disorders	9.68 (0.08)	10.16 (0.15)	9.78 (0.07)	147,785	.005
Renal failure	18.74 (0.10)	20.74 (0.20)	19.16 (0.09)	289,440	<.001
Chronic pulmonary disease	25.36 (0.11)	24.92 (0.21)	25.27 (0.10)	381,675	.061
Tobacco use	39.26 (0.16)	40.18 (0.30)	39.45 (0.14)	595,905	.007
Alcohol abuse	5.42 (0.05)	6.29 (0.11)	5.60 (0.05)	84,595	<.001
History of myocardial infarction	8.51 (0.07)	8.45 (0.13)	8.50 (0.06)	128,390	.650
History of CABG	9.39 (0.07)	8.94 (0.13)	9.30 (0.06)	140,425	.003
History of stroke	11.86 (0.07)	12.24 (0.14)	11.94 (0.07)	180,405	.017
Insurance carrier					.026
Medicare	70.35 (0.15)	69.73 (0.30)	70.22 (0.13)	1,036,055	
Medicaid	6.39 (0.08)	6.90 (0.15)	6.50 (0.07)	95,865	
Private insurance	20.70 (0.14)	20.73 (0.28)	20.71 (0.12)	305,515	
Self-pay	2.56 (0.05)	2.64 (0.09)	2.58 (0.04)	38,025	
Median household income					.053
0^th^–25^th^ percentile	27.47 (0.31)	28.45 (0.59)	27.68 (0.27)	411,525	
26^th^–50^th^ percentile	27.41 (0.25)	28.44 (0.45)	27.63 (0.21)	410,765	
51^st^–75^th^ percentile	24.82 (0.24)	23.37 (0.42)	24.51 (0.20)	364,400	
76^th^–100^th^ percentile	20.30 (0.33)	19.74 (0.60)	20.18 (0.28)	300,105	
Census division of hospital					.998
New England	5.30 (0.22)	5.21 (0.52)	5.28 (0.16)	79,720	
Middle Atlantic	14.25 (0.37)	13.72 (0.79)	14.14 (0.28)	213,595	
East North Central	17.27 (0.38)	16.86 (0.86)	17.18 (0.28)	259,520	
West North Central	6.97 (0.23)	7.00 (0.54)	6.97 (0.16)	105,310	
South Atlantic	23.28 (0.43)	23.94 (1.03)	23.42 (0.31)	353,820	
East South Central	7.12 (0.29)	7.49 (0.71)	7.20 (0.20)	108,760	
West South Central	10.72 (0.29)	10.75 (0.63)	10.73 (0.21)	162,010	
Mountain	5.13 (0.20)	5.17 (0.44)	5.14 (0.14)	77,660	
Pacific	9.96 (0.25)	9.85 (0.56)	9.94 (0.19)	150,070	
Bed size of hospital					.178
Small	21.94 (0.37)	23.96 (0.82)	22.37 (0.29)	337,825	
Medium	30.85 (0.45)	29.61 (1.01)	30.59 (0.33)	462,000	
Large	47.21 (0.50)	46.44 (1.16)	47.05 (0.36)	710,641	
Location/teaching status of hospital					.030
Rural	11.19 (0.24)	11.08 (0.50)	11.17 (0.18)	168,675	
Urban non-teaching	22.92 (0.37)	20.39 (0.83)	22.38 (0.26)	338,100	
Urban teaching	65.88 (0.43)	68.54 (0.95)	66.45 (0.31)	1,003,690	
Patient location: NCHS urban–rural code					.830
Central: population > 1,000,000	23.67 (0.47)	22.65 (0.83)	23.45 (0.40)	353,180	
Fringe: population < 1,000,000	25.81 (0.49)	25.68 (0.87)	25.78 (0.42)	388,235	
Population 250,000–999,999	21.24 (0.51)	21.95 (0.93)	21.39 (0.44)	322,060	
Population 50,000–249,999	10.35 (0.31)	10.55 (0.56)	10.39 (0.27)	156,515	
Micropolitan	10.70 (0.19)	10.88 (0.38)	10.74 (0.16)	161,660	
Rural	8.23 (0.16)	8.29 (0.32)	8.25 (0.14)	124,160	
Weekend admission	20.75 (0.10)	20.73 (0.19)	20.75 (0.09)	313,360	.933

*Abbreviations:* CABG, coronary artery bypass graft; NCHS, National Center for Health Statistics; SE, standard error. All values are given as % (SE) unless otherwise indicated.

**Supplementary Table 1:** ICD-10 Diagnosis and Procedure Codes

	Dyslipidemia	E780–E785
Diagnoses	Smoking	F17200, F17201, F17203, F17208, F17209, F17210, F17211, F17213, F17218, F17219, F17220, F17221, F17223, F17228, F17229, F17290, F17291, F17293, F17298, F17299, O99330, O99331, O99332, O99333, O99334, O99335, Z720, Z87891
	History of myocardial infarction	I252
	History of coronary artery bypass graft	V45.81, Z95.1, Z95.81, Z95.9
	TIA/ischemic stroke	I63.X, I9781x, I9782x, G45x
	Hemorrhagic stroke	I61.X, I62.X
Procedures	Cardiac catheter ablation	02543ZZ, 02553ZZ, 02563ZZ, 02583ZZ, 02593ZZ, 025D3ZZ, 025F3ZZ, 025G3ZZ, 025H3ZZ, 025J3ZZ, 025K3ZZ, 025L3ZZ, 025M3ZZ, 025N3ZZ 025P3ZZ, 025Q3ZZ, 025R3ZZ, 025S3ZZ, 025T3ZZ, 025V3ZZ, 02523ZZ, 02544ZZ 02554ZZ, 02564ZZ, 02584ZZ 02594ZZ, 025D4ZZ, 025F4ZZ, 025G4ZZ, 025H4ZZ, 025J4ZZ, 025K4ZZ, 025L4ZZ, 025M4ZZ, 025N4ZZ, 025P4ZZ, 025Q4ZZ, 025R4ZZ, 025S4ZZ, 025T4ZZ, 025V4ZZ, 02524ZZ,02573ZK, 02573ZZ, 02574ZZ, 02584ZZ, 02574ZK
	Percutaneous left atrial appendage closure	02L73DK
	Surgical or open or epicardial left atrial appendage closure	02L70CK, 02L70DK, 02L70ZK, 02B70ZK, 02B70ZK, 02B73ZK, 02B74ZK,
		02L73CK, 02L73ZK
	Transcatheter aortic valve implantation	02RF3JZ, 02RF3KZ, 02RF37Z, 02RF38Z, 02RF37H, 02RF38H, 02RF3JH, 02RF3KH, 02RF48Z, 02RF4JZ, 02RF4KZ, X2RF332, X2RF432
	Transcatheter mitral valve repair	02QG3ZZ, 02QG4ZZ
	Percutaneous coronary intervention	02703ZZ, 02704ZZ, 02713ZZ, 02714ZZ, 02723ZZ, 02724ZZ, 02733ZZ, 02734ZZ, 02Q03ZZ, 02Q04ZZ, 02Q13ZZ, 02Q14ZZ, 02Q23ZZ, 02Q24ZZ, 02Q33ZZ, 02Q34ZZ, 0270346, 027034Z, 0270356, 027035Z, 0270366, 027036Z, 0270376, 027037Z, 02703D6, 02703DZ, 02703E6, 02703EZ, 02703F6, 02703FZ, 02703G6, 02703GZ, 02703T6, 02703TZ, 02703Z6, 0270446, 027044Z, 0270456, 027045Z, 0270466, 027046Z, 0270476, 027047Z, 02704D6, 02704DZ, 02704E6, 02704EZ, 02704F6, 02704FZ, 02704G6, 02704GZ, 02704T6, 02704TZ, 02704Z6, 0271346, 027134Z, 0271356, 027135Z, 0271366, 027136Z, 0271376, 027137Z, 02713D6, 02713DZ, 02713E6, 02713EZ, 02713F6, 02713FZ, 02713G6, 02713GZ, 02713T6, 02713TZ,02713Z6, 0271446, 027144Z, 0271456, 027145Z, 0271466, 027146Z, 0271476, 027147Z, 02714D6, 02714DZ, 02714E6, 02714EZ, 02714F6, 02714FZ, 02714G6, 02714GZ, 02714T6, 02714TZ, 02714Z6, 02714ZZ, 0272346, 027234Z, 0272356, 027235Z, 0272366, 027236Z, 0272376, 027237Z, 02723D6, 02723DZ, 02723E6,02723EZ, 02723F6, 02723FZ, 02723G6, 02723GZ, 02723T6, 02723TZ, 02723Z6, 0272446, 027244Z, 0272456, 027245Z, 0272466, 027246Z, 0272476, 027247Z,02724D6, 02724DZ, 02724E6, 02724EZ, 02724F6, 02724FZ, 02724G6, 02724GZ, 02724T6, 02724TZ, 02724Z6, 0273346, 027334Z, 0273356, 027335Z, 0273366,027336Z, 0273376, 027337Z, 02733D6, 02733DZ, 02733E6, 02733EZ, 02733F6, 02733FZ, 02733G6, 02733GZ, 02733T6, 02733TZ, 02733Z6, 02733ZZ, 0273446, 027344Z, 0273456, 027345Z, 0273466, 027346Z, 0273476, 027347Z, 02734D6, 02734DZ, 02734E6, 02734EZ, 02734F6, 02734FZ, 02734G6, 02734GZ, 02734T6, 02734TZ, 02734Z6, 02734ZZ

*Abbreviations:* ICD-10, International Classification of Diseases, 10^th^ Revision; TIA, transient ischemic attack. Adapted from Vincent et al.^7^

**Supplementary Table 2:** Odds of Undergoing Ablation Therapy in Atrial Fibrillation Admissions

	Odds Ratio	*P* Value	95% CI
Calendar year			
2017	1.00		
2018	1.14	.033	1.01–1.28
2019	1.10	.134	0.97–1.24
2020	1.24	.000	1.10–1.40
Age	0.98	.000	0.97–0.98
Elixhauser comorbidities	1.10	.000	1.08–1.11
Female sex	0.73	.000	0.70–0.76
Race			
White	1.00		
Black	0.61	.000	0.56–0.67
Hispanic	0.83	.000	0.76–0.91
Asian or Pacific Islander	0.82	.010	0.70–0.95
Native American	0.81	.194	0.59–1.11
Other	1.08	.463	0.87–1.34
CHA_2_DS_2_-VASc score ≥ 2 points	1.92	.000	1.78–2.07
Hypertension	0.76	.000	0.73–0.80
Diabetes	0.75	.000	0.72–0.79
Hyperlipidemia	1.08	.000	1.03–1.12
Obesity	1.05	.052	1.00–1.10
Peripheral vascular disorders	1.00	.925	0.94–1.07
Renal failure	0.93	.003	0.88–0.97
Chronic pulmonary disease	0.96	.057	0.92–1.00
Tobacco use	0.88	.000	0.85–0.92
Alcohol abuse	0.36	.000	0.32–0.41
History of myocardial infarction	0.92	.012	0.86–0.98
History of CABG	1.24	.000	1.16–1.31
History of stroke	0.89	.000	0.85–0.94
Insurance carrier			
Medicare	1.00		
Medicaid	0.66	.000	0.60–0.72
Private insurance	1.06	.052	1.00–1.12
Self-pay	0.34	.000	0.28–0.40
Median household income (median)			
0^th^–25^th^ percentile	1.00		
26^th^–50^th^ percentile	1.05	.096	0.99–1.12
51^st^–75^th^ percentile	1.15	.000	1.08–1.23
76^th^–100^th^ percentile	1.35	.000	1.25–1.45
Census division of hospital			
New England	1.00		
Middle Atlantic	1.60	.000	1.32–1.94
East North Central	0.97	.757	0.82–1.16
West North Central	0.89	.257	0.73–1.09
South Atlantic	1.63	.000	1.38–1.93
East South Central	1.11	.371	0.89–1.38
West South Central	1.89	.000	1.57–2.28
Mountain	1.39	.002	1.13–1.72
Pacific	1.19	.091	0.97–1.44
Bed size of hospital			
Small	1.00		
Medium	1.75	.000	1.50–2.03
Large	2.90	.000	2.53–3.33
Location/teaching status of hospital			
Rural	1.00		
Urban non-teaching	3.72	.000	2.56–5.39
Urban teaching	8.65	.000	6.07–12.34
Patient location: NCHS urban–rural code			
Central: population > 1,000,000	1.00		
Fringe: population < 1,000,000	1.05	.281	0.96–1.14
Population 250,000–999,999	0.87	.003	0.80–0.96
Population 50,000–249,999	0.94	.262	0.84–1.05
Micropolitan	1.44	.000	1.28–1.62
Rural	1.24	.000	1.11–1.38
Weekend admission	0.46	.000	0.43–0.49
Intercept	0.01	.000	0.01–0.02

*Abbreviations:* CABG, coronary artery bypass graft; CI, confidence interval; NCHS, National Center for Health Statistics. This table shows the results of multivariate logistical regression of ablation therapy in atrial fibrillation admissions, by year.

**Supplementary Table 3:** Logistic Regression Model for Inpatient Mortality

	Odds Ratio	*P* Value	95% CI
Calendar year			
2017	0.99	.902	0.88–1.11
2018	0.94	.266	0.83–1.05
2019	1.11	.087	0.99–1.25
2020	0.64	.000	0.52–0.80
Age	1.05	.000	1.04–1.06
Elixhauser comorbidities	1.66	.000	1.62–1.70
Female sex	0.73	.000	0.67–0.79
Race			
White	1.00		
Black	1.28	.001	1.10–1.48
Hispanic	1.16	.095	0.97–1.39
Asian or Pacific Islander	1.20	.239	0.88–1.64
Native American	1.20	.570	0.64–2.22
Other	1.05	.761	0.78–1.40
CHA_2_DS_2_-VASc score ≥ 2 points	0.87	.206	0.70–1.08
Hypertension	0.42	.000	0.38–0.47
Diabetes	0.70	.000	0.64–0.77
Hyperlipidemia	0.57	.000	0.52–0.62
Obesity	0.44	.000	0.39–0.50
Peripheral vascular disorders	0.71	.000	0.63–0.81
Renal failure	1.02	.613	0.93–1.13
Chronic pulmonary disease	0.82	.000	0.75–0.90
Tobacco use	0.75	.000	0.69–0.82
Alcohol abuse	0.55	.000	0.45–0.68
History of myocardial infarction	0.99	.881	0.86–1.14
History of CABG	0.81	.006	0.70–0.94
History of stroke	1.22	.000	1.10–1.36
Insurance carrier			
Medicare	1.00		
Medicaid	1.40	.001	1.14–1.73
Private insurance	1.03	.743	0.88–1.20
Self-pay	1.46	.031	1.04–2.05
Median household income			
0^th^–25^th^ percentile	1.00		
26^th^–50^th^ percentile	1.01	.885	0.91–1.12
51^st^–75^th^ percentile	0.96	.464	0.85–1.08
76^th^–100^th^ percentile	0.84	.020	0.73–0.97
Census division of hospital			
New England	1.00		
Middle Atlantic	1.20	.130	0.95–1.51
East North Central	0.90	.399	0.71–1.14
West North Central	1.01	.965	0.76–1.33
South Atlantic	1.12	.312	0.90–1.41
East South Central	1.40	.011	1.08–1.81
West South Central	1.23	.091	0.97–1.57
Mountain	0.91	.519	0.69–1.21
Pacific	1.35	.017	1.05–1.73
Bed size of hospital			
Small	1.00		
Medium	0.96	.472	0.85–1.08
Large	1.06	.300	0.95–1.18
Location/teaching status of hospital			
Rural	1.00		
Urban non-teaching	1.12	.256	0.92–1.37
Urban Teaching	1.22	.040	1.01–1.47
Patient location: NCHS urban–rural code			
Central: population > 1,000,000	1.00		
Fringe: population < 1,000,000	1.06	.344	0.94–1.21
Population 250,000–999,999	1.22	.002	1.08–1.39
Population 50,000–249,999	1.23	.008	1.06–1.44
Micropolitan	1.29	.013	1.06–1.58
Rural	1.36	.001	1.12–1.64
Weekend admission	1.08	.120	0.98–1.19
Intercept	0.00	.000	0.00–0.00

*Abbreviations:* CABG, coronary artery bypass graft; CI, confidence interval; NCHS, National Center for Health Statistics.
